# Validation of the Electrophilic Allergen Screening Assay for Detection of Key Event 1 of the Skin Sensitization Adverse Outcome Pathway

**DOI:** 10.3390/toxics14060511

**Published:** 2026-06-11

**Authors:** Emily N. Reinke, Elijah J. Petersen, John D. Gordon, Rick Uhl, Valerie H. Adams, Diego Rua, Victor J. Johnson, Gary R. Burleson, Judy Strickland, Robert Gutierrez, Catherine S. Sprankle, Tripp LaPratt, David M. Lehmann, Dori R. Germolec, Nicole C. Kleinstreuer

**Affiliations:** 1 Inotiv, Inc., Morrisville, NC 27560, USA; judystrickland@frontier.com (J.S.); tripp.lapratt@gdit.com (T.L.); 2 GDIT, Inc., Falls Church, VA 22042, USA; 3 National Institute of Standards and Technology, Gaithersburg, MD 20899, USA; 4 U.S. Consumer Product Safety Commission, Rockville, MD 20850, USA; jgordon@cpsc.gov (J.D.G.);; 5 Defense Centers for Public Health—Aberdeen, Aberdeen, MD 21005, USA; 6 DRX2 Consulting LLC, Washington, DC 20001, USA; 7 Burleson Research Technologies, Inc., Morrisville, NC 27506, USAgburleson@brt-labs.com (G.R.B.); 8 Intertox, LLC, Seattle, WA 98101, USA; dlehmann@intertox.com; 9 National Institute of Environmental Health Sciences, Durham, NC 27709, USA; germolec@niehs.nih.gov; 10 Division of Program Coordination, Planning, and Strategic Initiatives, National Institutes of Health, Bethesda, MD 20892, USA

**Keywords:** hapten-carrier formation, haptenation, skin sensitization, Key Event 1, KE1, adverse outcome pathway, molecular initiating event, validation, transferability, reproducibility, performance standards

## Abstract

The electrophilic allergen screening assay (EASA) uses small-molecule probes as surrogates for skin proteins to measure hapten protein carrier complex formation, Key Event (KE) 1 in the adverse outcome pathway for skin sensitization. Although multiple assays are accepted for this purpose, the EASA has higher throughput and needs less specialized equipment than other commonly used KE1 assays. This validation study assessed transferability of the EASA to naïve laboratories and within- and between-laboratory reproducibility. The predictive capacity of the assay in relation to reference data both from the murine local lymph node assay (LLNA) and, where available, human predictive patch tests, was estimated. The validation study was conducted using performance standards developed for methods that are under evaluation for inclusion in relevant test guidelines. The within- and between-laboratory reproducibility were cumulatively 96% and 85%, respectively. These scores exceeded the performance standard thresholds of 80%. Compared to reference LLNA data, the cumulative EASA results from the four laboratories had an overall sensitivity of 87%, specificity of 76%, and accuracy of 83%. The consensus results among the four laboratories had an overall sensitivity of 85%, specificity of 80%, and accuracy of 83%. For human reference data, the cumulative EASA results had an overall sensitivity of 81%, specificity of 76%, and accuracy of 70%. The EASA predicted sensitizers very well, although it had a slightly higher rate of misclassifying some negative test chemicals as positive with a specificity below the performance standards criterion of 80%. Our findings support further exploring use of the EASA in defined approaches to identify potential skin sensitizers.

## 1. Introduction

In a significant portion of the human population, dermal exposure to certain chemicals can lead to skin sensitization and may progress to development of allergic contact dermatitis, a long-term and potentially debilitating disease [1,2]. Allergic contact dermatitis is a major concern for the cosmetic, agrochemical, and industrial chemical sectors [3]. For this reason, it is important to determine the potential for a product or ingredient to be a skin sensitizer through testing prior to availability on the open market or regulatory approval. Historically, skin sensitization has been assessed by either a guinea pig maximization test or the murine local lymph node assay (LLNA), which are in vivo models accepted by many regulatory agencies worldwide for identifying skin sensitization hazards and used as benchmarks for hazard classification [4,5]. With advancements in characterizing toxicological molecular mechanisms and societal preference toward reducing animal testing or eliminating it entirely, regulatory agencies worldwide have pursued non-animal methods for estimating the potential health effects of chemicals. These approaches are driven by regulations either restricting animal use (e.g., European Union) [6,7] or directing some agencies to consider alternatives to animal testing (e.g., United States) [8].

Skin sensitization induced by reactive chemicals is a multistep process described in a well-established adverse outcome pathway [9]. Small molecules capable of penetrating the skin barrier will interact with biomolecules including proteins driven by their inherent electrophilic reactivity. Four key events (KEs) are necessary for initiation, induction, and elicitation of skin sensitization. KE1 is the molecular initiating event of covalent binding of small-molecular-weight electrophilic chemicals to nucleophilic centers present in skin proteins. The nucleophilic sites are typically in cysteine or lysine residues. The covalent binding of reactive chemicals to skin proteins results in hapten protein carrier complex formation, also known as haptenation. This complex results in the formation of a neo-epitope that the immune system can recognize following processing and presentation by dendritic cells. KE2 is induction of inflammatory responses and cytoprotective pathways such as antioxidant responses in keratinocytes. KE3 is mobilization and activation of dendritic cell populations and migration to the lymph nodes, marked via the translocation of specific cellular membrane proteins to the cell surface. Finally, KE4 is T-cell activation and proliferation. Together, these KEs culminate in a misguided immune response to the hapten-carrier protein complex in the skin, resulting in the adverse outcome of allergic contact dermatitis.

The electrophilic allergen screening assay (EASA) was originally developed by Chipinda et al. [10,11] at the National Institute of Occupational Safety and Health as a cuvette-based screening assay to identify potential skin sensitizers. The EASA was later modified to a 96-well format to increase throughput, the number of control measurements, and accessibility of the assay [12]. The EASA addresses KE1 by measuring the covalent binding of test substances with a protein/peptide surrogate, chemical probes that mimic the protein/peptide–chemical interaction. The use of chemical probes in the EASA is predicated on the concept of a hard–soft acid-base, wherein “soft” electrophiles bind more tightly with “soft” nucleophiles and “hard” electrophiles bind more tightly with “hard” nucleophiles. The EASA probes act as surrogates for lysine- or cysteine-containing peptides [13], enabling the assay to interrogate the potential for chemicals to form covalent bonds with proteins. The EASA is thereby similar to other tests described in Test Guideline (TG) 442C issued by the Organisation for Economic Co-operation and Development (OECD) such as the direct peptide reactivity assay (DPRA) and the amino acid derivative reactivity assay (ADRA) [14]. Compared to those assays, the 96-well-plate format version of the EASA utilizes less specialized equipment (e.g., common plate reader), increasing its potential to be used in a wider range of laboratory settings.

In response to a nomination from the National Institute of Occupational Safety and Health and interest from several U.S. research and regulatory agencies, the Interagency Coordinating Committee on the Validation of Alternative Methods agreed to evaluate the EASA as a screening assay for predicting the skin sensitization hazard for chemicals.

This paper describes the validation study of the EASA as an alternative to other OECD TG 442C assays for assessment of KE1 in the skin sensitization adverse outcome pathway [14]. A specific goal of this study was to determine whether the performance of the EASA supports its inclusion into TG 442C. The validation study was conducted according to the OECD performance standards for TG 442C assays [15], and assessments of within- and between-laboratory reproducibility and accuracy for the classification of hazards were evaluated relative to targets stated in the performance standards. The performance standards were updated in 2022 shortly after the EASA in-laboratory testing was completed [16]. The update revised the list of chemicals used for the performance evaluation but did not change the EASA validation procedure. Some of the results described here were first published in a NICEATM report [17]. This manuscript provides significant additional experimental results, discussion, and data analysis that more fully evaluate the robustness of the results described in the government document.

## 2. Materials and Methods

### 2.1. Principle of the EASA

The EASA predicts a chemical’s tendency to bind to cysteine and lysine residues on a protein as a representation of its broader ability to bind skin proteins. The EASA protocol used in the validation study consists of two separate spectrophotometric assays using two different chemical probes. The probe 4-nitrobenzenethiol (NBT) represents binding of a test chemical to cysteine, while pyridoxylamine hydrochloride (PDA) is used as a probe to represent binding of a test chemical to lysine. EASA protocols describe how to evaluate test chemical binding by measuring chemical probe depletion. Probe depletion is measured as changes in absorbance of NBT at 412 nm and fluorescence of PDA at an excitation wavelength of 324 nm and an emission wavelength of 398 nm. The original EASA protocols also included a PDA absorbance assay at 324 nm, but this assay was not included in the validation study as it did not provide any predictive advantage beyond those of the other two endpoints [12].

The output metric of the EASA is the relative depletion of the chemical probe for each assay as compared to negative controls (without test chemicals), calculated using a modified *t*-test to account for variability within a plate for each given experiment [12]:

$$t=\frac{\left(\bar{NC}-\bar{S})-(\bar{TC}-\bar{{TC}_{B}}\right)}{\sqrt{\frac{{sd}_{NC}^{2}}{n_{NC}}+\frac{{sd}_{S}^{2}}{n_{S}}+\frac{{sd}_{TC}^{2}}{n_{TC}}+\frac{{sd}_{{TC}_{B}}^{2}}{n_{{TC}_{B}}}}}$$

Abbreviations: n = number of replicates; NC = negative control; S = Blank (solvent system only); sd = standard deviation; TC = test chemical; TC_B_ = Test chemical blank (test chemical and solvent system only). Bar over an abbreviation represents the mean of the test group.

**Figure 1:**
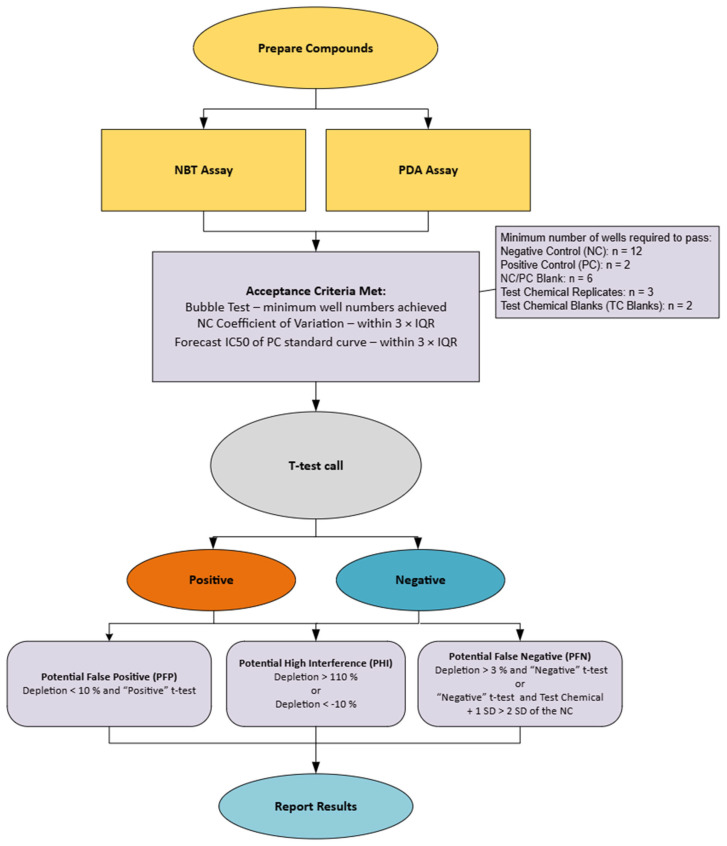
EASA decision tree and acceptance criteria. IQR = Interquartile range; SD = standard deviation; 3 × IQR = three times the IQR; 1SD = one times the standard deviation; 2SD = two times the standard deviation. Modified and reprinted with permission from [17].

The degrees of freedom are calculated using the Welch–Satterthwaite approximation. The results from the *t*-test approach were shown to be in close agreement with a Bayesian analysis approach [12]. A result for a test chemical is considered positive if a test passes the acceptance criteria described in Section 2.6, and the test chemical exhibits a statistically significant difference from the negative control (NC). A decision tree is provided in Figure 1 describing the decision criteria. In addition to the *t*-test, the data analysis procedure includes flags for potential false positives or negatives and for high interference to help in determining the validity of a test. The performance of test chemicals using thresholds at 3%, 5%, 10%, and 20% depletion for each probe was also evaluated using data from Laboratory 1 for comparison to the *t*-test approach.

### 2.2. General Conduct of the EASA

**Figure 2:**
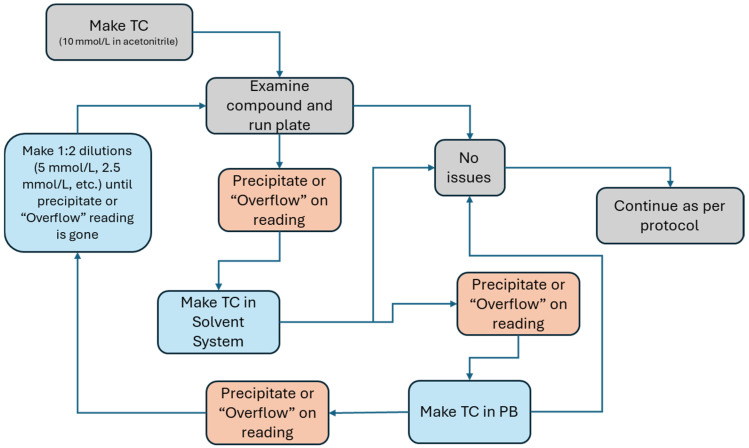
Test chemical assessment flow chart for selecting appropriate solvents. Abbreviations: TC = test chemical; PB = phosphate buffer. Modified and reprinted with permission from [17].

The validation study used a modification of the EASA 96-well-plate format protocol [12]. Briefly, on the day of testing, each test chemical was weighed and prepared as a 10 mmol/L stock solution in the appropriate solvent system (SS). This was typically 50% acetonitrile (ACN) and 50% 0.1 mol/L phosphate buffer (pH 7.4 ± 0.2) unless there were solubility incompatibilities, at which point a decision tree was used to determine an appropriate alternative solvent (Figure 2). Appropriate positive control (PC) solutions (3 mmol/L benzyl bromide in ACN for the NBT assay and 1 mmol/L glutaraldehyde in ACN for the PDA assay) were similarly prepared. A plate reader with appropriate absorbance and fluorescence capabilities was turned on and allowed to stabilize before plates were set up.

The following is a list of reagents and materials used in this study: acetonitrile (HPLC-grade), pyridoxylamine (analytical standard, ≥98%), 4-nitrobenzenethiol (technical grade, 80%), benzyl bromide (reagent grade, 98%), and VIEWseal multi-well plate sealers were from Sigma-Aldrich (St. Louis, MO, USA); glutaraldehyde (50% certified) was from Thermo Fisher Scientific (Waltham, MA, USA); and Bio-One UV-Star 96 well UV spectroscopy microplates were from Greiner (Kremsmünster, Austria).

**Figure 3:**
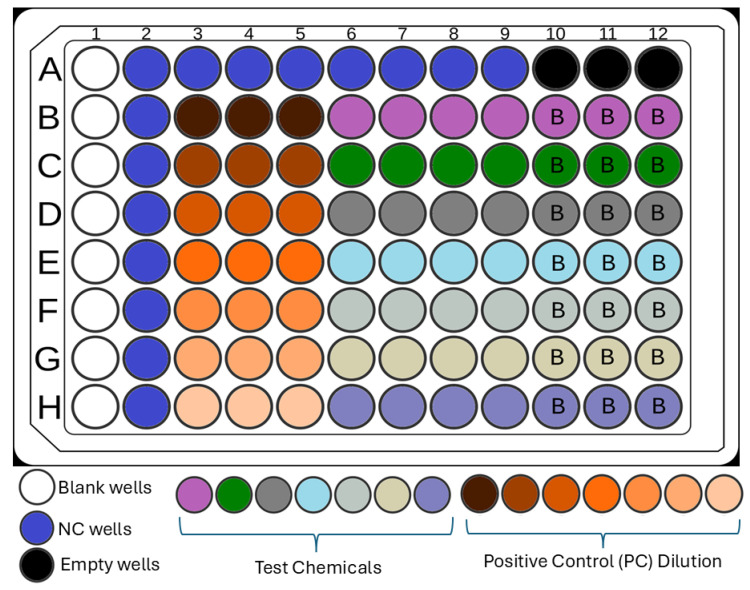
Plate map for the 96-well EASA. Abbreviations: NC = negative control, PC = positive control. B = Test Chemical blank. Figure modified and reprinted with permission from [12].

The plate map in Figure 3 shows the setup of each plate, including test chemical(s), PCs, NCs (SS and test probe only), and blanks (SS only (Blanks) or SS plus test chemical but without probe (Test chemical blanks)). Empty wells have no added liquids. This specific plate layout was important to facilitate data input into the data calculator spreadsheet.

For the NBT assay, a 0.125 mmol/L NBT working solution was made by 1:8 dilution of a 1 mmol/L NBT stock solution in SS. Plates were then loaded as follows:

1. ACN (40 µL) was added to appropriate Blank wells.
2. ACN (40 µL) was added to appropriate NC wells.
3. Benzyl bromide stock solution in ACN (40 µL) was added to appropriate PC wells in Row B followed by a 1:2 serial dilution in each row down to Row H.
4. Test chemical stock solution (40 µL) was added to appropriate test chemical and test chemical blank wells (Columns 6–10 omitting Row A).
5. SS (160 µL) was added to wells without added test chemicals or the PC (all rows of Column 1, and Rows B through H of Columns 10, 11, and 12).
6. At this point, a red light was turned on, and standard lab lights were turned off.
7. Immediately after starting the reaction timer, 160 µL NBT working solution was added to wells in Columns 2–9. The plate was sealed when all wells were filled.

Plate setup for the PDA fluorescence assay was done similarly to the NBT assay, with the following key changes. Glutaraldehyde was used as the PC. For the probe, a 0.008 mmol/L PDA working solution was made by adding 200 µL of 1 mmol/L PDA stock to 25 mL SS. Step 6 (lab lights being turned off) was omitted. Given different sensitivities of plate readers for fluorescence signal, PDA was titrated from the recommended 0.008 mmol/L if the signal approached the maximum for the plate reader. The optimal PDA concentration was targeted to be at least 20% below maximal signal and may be unique for each instrument and laboratory.

For all assays, absorbance or fluorescence was read at nominal timepoints of 5 min, 20 min, 35 min, and 50 min. Percent depletion at each timepoint was calculated as detailed above, but only the 50 min timepoint was used to determine the result of a test. Prior to the first timepoint, a “bubble” test was conducted at 680 nm to detect interference from bubbles or test chemicals (see Section 2.6 below).

### 2.3. Chemicals Tested

Table 1 lists the 20 reference chemicals tested in the study, 12 of which were used to assess within-laboratory reproducibility. All 20 chemicals were used to test between-laboratory reproducibility. Test chemicals were provided by MRIGlobal, chemistry contractor for the National Institute of Environmental Health Sciences Division of Translational Toxicology. A chemical safety officer at each facility was identified to receive test chemicals and complete chemical information (i.e., physical state, weight/volume, specific density for liquids, and storage instructions). All chemicals were coded at the NTP Chemical Repository, with testing facility safety officers instructed on cataloging, storage, and handling for each chemical. Sealed safety data sheets were provided for all 20 test chemicals, with a separate code-breaker spreadsheet emailed to safety officers to facilitate development of a chemical safety plan. Chemical disposal was under the purview of the safety officer at each facility, following local regulations for appropriate disposal.

### 2.4. Study Plan

Four U.S. government-sponsored laboratories (Laboratories 1–4) participated in the testing. These were, in alphabetical order: sponsoring agency U.S. Consumer Product Safety Commission (CPSC) partnered with the National Institute of Standards and Technology (NIST); U.S. Department of Defense (Army Public Health Center/Defense Center for Public Health—Aberdeen); U.S. Food and Drug Administration (Center for Devices and Radiological Health); and National Institute of Environmental Health Sciences (testing performed at contract laboratory Burleson Research Technologies, Inc., Morrisville, NC, USA). The study was coordinated by NICEATM, with scientists from the U.S. Food and Drug Administration and the U.S. Environmental Protection Agency serving on the validation management team.

The study included a prevalidation method-transfer phase and a validation phase. In the prevalidation phase, three of the four participating laboratories attended on-site training on the conduct of the assay with CPSC/NIST test developers at NIST. Following the in-person training, each participating lab conducted PC and NC (solvent) tests; 10 plates with only the NC and PC wells filled were evaluated using both the NBT absorbance and PDA fluorescence assays. In addition to serving as the technical transfer portion of the study, this provided each lab with a reference database of PC and NC data for the development of test acceptance criteria. Initial data from Laboratory 3 showed higher-than-expected variability for the PDA assay NC. Experiments were performed by adjusting the PDA concentration and plate reader gain to assess if the precision could be improved.

The validation phase included assessments of within-laboratory and between-laboratory reproducibility [15]. Each chemical was tested in two independent repetitions for both the EASA probes. For each probe, if the first two repetitions were not concordant, a third repetition was conducted to determine the outcome. Concordance among 2 of 2 or 2 of 3 repetitions constituted a qualified test (QT). Three QTs were completed on each chemical that was used to test the within-laboratory reproducibility (Table 1). Overall reproducibility was calculated as an average of the NBT and the PDA assay reproducibility among all QTs. To assess between-laboratory reproducibility, the remaining eight chemicals had one QT each completed. All 20 of the chemicals were evaluated for predictive capacity by comparing to reference LLNA data provided in the performance standards and reference human data available in Annex 2 of the Supporting Documents for Guideline 497 [18]. Predictive capacity of a test method for hazard classification considered the number of true positive (TP), true negative (TN), false positive (FP), and false negative (FN) outcomes relative to reference data. Accuracy, sensitivity, and specificity were calculated as follows:

$$Accuracy\left(\%\right)=\left[\frac{TP+TN}{TP+TN+FP+FN}\right]\times 100$$

$$Sensitivity(\%)=[\frac{TP}{TP+FN}]\times 100$$

$$Specificity(\%)=[\frac{TN}{TN+FP}]\times 100$$

Cumulative performance of the assay was calculated using the total number of predictions for all 20 chemicals for the four performance measures (TP, TN, FP, FN) across all laboratories (resulting in a total of 80 predictions).

### 2.5. Data Management

The test method developers at CPSC and NIST designed check sheets for use by participating labs for preparation of reagents, study conduct, and data analysis (see [17]). Data were entered into Microsoft Excel data calculator files, which were prepared and locked by the test method developers. A separate workbook was provided for tracking quality control (QC) data. QC values were copied and pasted between the two files as specified in the worksheet calculations to determine if an assay met acceptance criteria and to make determinations on test chemical outcomes. All electronic worksheets were stored on servers according to the study protocol and/or applicable standard operating procedures (SOPs) of each individual participating laboratory, and electronic copies were provided to NICEATM for final collation and evaluation. Paper check sheets were stored as specified in the study protocol and/or applicable SOPs of each individual testing facility.

### 2.6. Acceptance Criteria

Acceptance criteria were established in advance to determine whether a run was successful. While all records were available to allow for analysis of failed run occurrence and frequency, only successful runs were included in statistical analyses and in the evaluation of the validation study. One criterion for assessing the success of a run was passing of a “bubble test,” conducted during the first 5 min of incubation. This consisted of a reading at 680 nm to determine if any physical interference, such as bubbles or precipitate, was present in the well. According to the criterion, a single well with an absorbance above 0.085 was considered an outlier and was excluded from analysis. This value is higher than the threshold of 0.079 used in [12], and was based on new data from multiple laboratories during the initial phase of this interlaboratory study. If all test chemical wells exceeded 0.085, they were not excluded, as this is likely due to the use of colored test chemicals. Figure 1 summarizes the bubble test criteria needed for a successful run.

In addition to passing the bubble test, additional criteria for a successful run were for each plate to have control values within three times the interquartile range defined by the prevalidation historical control data established by each laboratory. For the NC, the coefficient of variation for the wells in the run was calculated and compared to the range of historical control data. For the PC, the forecast half-maximal inhibitory concentration (IC_50_) was determined from the PC standard curve and compared to the range of historical control data.

## 3. Results

### 3.1. General Issues Encountered During the Conduct of the Assay

The prevalidation of the EASA was launched in April 2019, with labs completing their 10 prevalidation plates by fall of 2019. The prevalidation revealed within-plate inconsistencies in fluorescence readings in Laboratory 3, specifically higher fluorescence output on the right side of the plate compared to the left, which needed additional investigation (Supplemental Figure S1). The outcome of the investigation resulted in adjustments to the protocol to allow for titration of the PDA concentration based on plate reader sensitivity and the likelihood of overflow readings in adjacent wells. Use of a decreased PDA concentration in Laboratory 3 eliminated this inconsistency; reducing the PDA concentration by 50% yielded a decrease of approximately 50% in the average PDA coefficient of variation for the NCs. Additional testing in Laboratory 1 of seven of the test chemicals revealed that varying the PDA and NBT concentrations between 50% and 200% of the nominal concentration did not impact the calls for one QT (Supplemental Table S1). This is likely a result of the probe concentration being substantially greater than the test chemical concentration. This finding revealed that the assay results are insensitive to large changes in the probe concentration.

Laboratories received reference chemicals in February 2020, but testing was paused until summer due to the COVID-19 pandemic. Laboratory 2 completed testing reference chemicals by the end of August 2020. Prevalidation testing needed to be repeated at Laboratory 3 because of the within-plate inconsistencies in fluorescence output noted during the prevalidation phase.

Pandemic-related supply chain issues contributed to delays in testing at Laboratories 1 and 4. Differences in fluorescence background in newer 96-well plates compared to older ones caused runs to fail when assessed according to historical prevalidation data. This difficulty was resolved by laboratories screening older plates for similar fluorescence background levels and then sharing acceptable lots of plates with other laboratories. NBT was also found to have lot-to-lot variability at both Laboratories 1 and 3, with different lots both above and below the QC parameters for the NC wells for the historical prevalidation data. The lead lab determined that, in the future, reagents should be assessed for concordance with the QC parameters. The lead lab also recommended that reagents found to be out of specifications should have new QC parameters determined specific to the current lot/time period, and that these steps should be taken upon receipt of new lots (specifically for plates and control reagents) or after a long gap in testing.

Delays for reference chemical testing at Laboratories 1 and 3 occurred in March 2021 when it was found that some of the test chemicals had expired. The NTP Chemical Repository purchased fresh chemicals and shipped new aliquots of coded reference chemicals to Laboratories 1, 3, and 4 in June 2021. Reagent expiration may have contributed to difficulties encountered by Laboratory 2 in meeting the test acceptance criteria established by the prevalidation study. Other potential contributing factors include the use of incorrect probe or PC concentration, pipettor calibration errors, and plate lot variability. Laboratory 3 experienced air conditioning issues resulting in a high ambient temperature in the laboratory, which appeared to affect NBT but not PDA assay results. NBT plates failed as a result of the IC_50_ not passing QC. Minimum and maximum chamber temperatures during a run were tracked as part of the normal data acquisition process of the plate reader utilized (Molecular Devices SpectraMax^®^ i3x). These were charted and it was found that failed NBT plates had chamber temperatures that ranged from 26 °C to 31.5 °C, while chamber temperatures for passing plates ranged from 20.5 °C to 27.5 °C (Supplemental Figure S2A). This was not the case for PDA plates, for which no failures were observed under temperatures ranging from 21 °C to 31.5 °C (Supplemental Figure S2B). This indicates that, at a minimum, the kinetics for the benzyl bromide PC for NBT are affected by temperature. Further assessment would be necessary to determine if NBT stability is also affected, although the NC mean was unaffected by chamber temperature.

### 3.2. Transferability Assessment

Assessment of transferability was accomplished during the prevalidation phase through in-person training and the completion of 10 prevalidation NC/PC plates for NBT absorbance and PDA fluorescence. This stage also allowed for optimization of the test protocol and calculation worksheets based on feedback from each of the participating laboratories.

Prior to testing the reference chemicals in the EASA, each lab established baseline QC parameters for evaluating the performance of the assays. These summary data included upper and lower limits for Blanks, NCs, and NC coefficients of variation (COVs). Midpoint, forecast IC_50_, and dilution series upper and lower limits were also established for the PC. Baseline parameter data for all laboratories are provided in Supplemental Tables S2–S5. Results of this phase confirmed that each facility had achieved sufficient proficiency in the assay to proceed to the later phases of the study.

### 3.3. Within-Laboratory Reproducibility

Each laboratory completed three QTs on each of 12 reference chemicals to assess within-laboratory reproducibility (WLR) as described in Section 2.4. Table 2 lists the chemicals tested and the overall EASA calls obtained in each laboratory during this phase of testing. Individual run calls for each probe and test chemical for all laboratories are provided in Supplemental Table S6.

The average WLR for EASA calls from all four laboratories based on completed QTs was 98%, with a WLR of 94% at Laboratory 1 and 100% at the other three labs. These exceed the benchmark of 80% specified in OECD performance standards for in vitro skin sensitization test methods [15]. The probe-specific WLRs were 95% and 97% for NBT and PDA, respectively. Results for salicylic acid were considered inconclusive in three of the four labs, while the fourth lab obtained a clear negative result. While each individual PDA assay resulted in a negative call, these results were considered inconclusive because this test chemical was run in these labs at a concentration lower than that prescribed by the protocol to avoid fluorescence interference. A similar issue was found with 4-(methylamino) phenol hemisulfate, for which the same three labs were not able to obtain conclusive calls with the PDA probe. However, because the test chemical was consistently positive in the NBT, it was correctly classified by the EASA. Two labs also misclassified benzyl alcohol as positive, when compared to the reference LLNA data in the performance standards [15], with a third having a positive result in one of their QTs. These mixed results are consistent with the mixed results obtained for these three chemicals in the DPRA validation study [19].

#### 3.3.1. Laboratory 1

Laboratory 1 conducted 37 NBT assays and 33 PDA assays. Of these assays, two NBT and six PDA plates failed the forecast IC_50_ criterion for the PC. All other plates passed and were included in the WLR assessment. There were no failures on an individual chemical basis due to lack of adequate replicates.

Laboratory 1 had a WLR rate of 94% for the 12 WLR assessment chemicals. For the individual assays, the WLR for NBT was 92% and was 93% for PDA. The only chemical misclassified relative to the in vivo prediction was the weak sensitizer ethyl acrylate.

#### 3.3.2. Laboratory 2

Laboratory 2 conducted 19 NBT assays and 25 PDA assays. Of these assays, one NBT and three PDA plates failed the forecast IC_50_ criterion for the PC. All other plates passed and were included in the WLR assessment. There were five failures on an individual chemical basis due to a lack of adequate replicate wells for that chemical on a plate.

Laboratory 2 had a WLR rate of 100% on the basis of completed QTs, both overall and for the individual assays. Two chemicals were misclassified, the weak sensitizer ethyl acrylate and the nonsensitizer benzyl alcohol.

#### 3.3.3. Laboratory 3

Laboratory 3 conducted 27 NBT assays and 32 PDA assays. Of these assays, four NBT plates failed the forecast IC_50_ criterion for the PC, and two PDA plates failed the NC COV criteria. All other plates passed and were included in the WLR assessment. There were no failures on an individual chemical basis.

Laboratory 3 had a WLR rate of 100% on the basis of completed QTs. For the individual assays, the WLR for NBT was 92% and for PDA was 100%. The only chemical misclassified relative to the in vivo prediction was the weak sensitizer ethyl acrylate.

#### 3.3.4. Laboratory 4

Laboratory 4 conducted 23 NBT assays and 34 PDA assays. All plates passed and were included in the WLR assessment. There were no failures on an individual chemical basis.

Laboratory 4 had a WLR rate of 100% on the basis of completed QTs. For the individual assays, the WLR for NBT was 94% and for PDA was 94%. There were two chemicals misclassified, the weak sensitizer ethyl acrylate and the nonsensitizer benzyl alcohol.

### 3.4. Between-Laboratory Reproducibility

Each laboratory tested 20 reference chemicals to assess between-laboratory reproducibility (BLR). Table 3 lists the chemicals tested, and the overall EASA calls obtained in each laboratory during this phase of testing. For the eight chemicals identified in Table 1 as being tested only for the BLR phase, results were based on one QT. For the 12 chemicals tested in the WLR phase, BLR was based on the consensus of three QTs.

The four testing facilities produced discordant classifications for three of the 20 test chemicals: salicylic acid, benzyl alcohol, and dihydroeugenol (Supplemental Table S7). Thus, overall BLR was 85%. This exceeds the benchmark of 80% specified in OECD performance standards for in vitro skin sensitization test methods [15]. Laboratories 1, 3, and 4 obtained inconclusive outcomes for salicylic acid, due to the need to dilute the test article below protocol specifications to resolve fluorescence interference with the PDA probe. The EASA results for benzyl alcohol were mixed, with two negatives and two positives for the labs. These results are similar to results obtained for this compound during the DPRA validation [19]. Mixed results were also obtained for the prohapten dihydroeugenol, which is a nonsensitizer in human tests [20]. In the EASA, three labs obtained a negative classification, but Laboratory 2 obtained a positive call. These mixed results are consistent with conflicting results for this chemical from validation of a number of in vitro skin sensitization assays, including the DPRA, ADRA, the human cell line activation test, and KeratinoSens [21].

### 3.5. Predictive Capacity Results

Predictive capacity for the EASA was determined by calculating sensitivity, specificity, and accuracy for the 20 reference chemicals relative to LLNA data. Performance of the EASA against human data was also assessed for 13 reference chemicals. Table 3 provides a summary of the four participating lab test outcomes for the 20 test chemicals along with a comparison to reference data.

**Figure 4:**
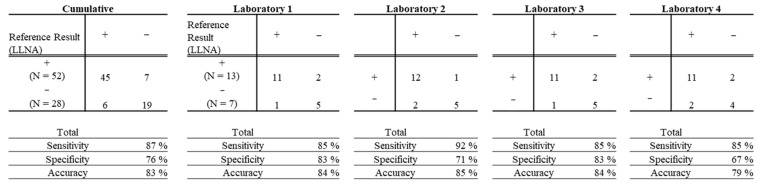
Predictive capacity of the EASA when compared to LLNA reference data. Modified and reprinted with permission from [17].

Performance standards for assessment of skin sensitization test methods similar to the DPRA and ADRA [15] provide benchmarks of 80% for sensitivity, specificity, and accuracy relative to reference data. Accuracy of the EASA compared to the LLNA was 84% for Laboratory 1, 85% for Laboratory 2, 84% for Laboratory 3, and 79% for Laboratory 4, with a cumulative accuracy of 83% (Figure 4). Sensitivity was 85% for Laboratories 1, 3, and 4, and 92% for Laboratory 2. Specificity for the four laboratories was 83%, 71%, 83%, and 67% respectively. The cumulative sensitivity was 87%, and cumulative specificity was 76%. The consensus results among the four laboratories had an overall sensitivity of 85%, specificity of 80%, and accuracy of 83%.

The data from Laboratory 1 data were also evaluated using a threshold approach, where different percent depletion amounts (3%, 5%, 10%, and 20%) of the chemical probe were used as a cut-off for a positive outcome. Use of these thresholds yielded identical concordance results for thresholds of 3%, 5%, and 10% compared to the *t*-test approach. Using a threshold value of 20%, the concordance decreased for two chemicals which became false negative results.

**Figure 5:**
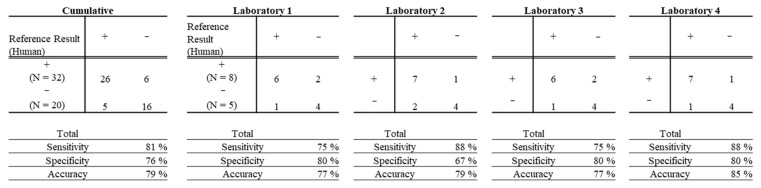
Predictive capacity of the EASA when compared to human predictive patch test data. Modified and reprinted with permission from [17].

When compared against human data, the accuracy of the EASA was 77% for Laboratories 1 and 3, 79% for Laboratory 2, and 85% for Laboratory 4, with a cumulative accuracy of 79% (Figure 5). Sensitivity was 75% for Laboratories 1 and 3, and 88% for Laboratories 2 and 4, for a cumulative sensitivity of 81%. Specificity was 80% for Laboratories 1, 3, and 4, and 67% for Laboratory 2, for a cumulative specificity of 76%.

## 4. Discussion

There is an increasing international need for validation of non-animal methods to predict the toxicity of chemicals, driven by regulatory directives and economic and ethical concerns [6,7,8]. Assays measuring the tendency for a chemical to bind to skin proteins can be used in defined approaches for skin sensitization, which are becoming increasingly accepted as alternatives to animal use for identifying potential sensitizers [22]. Defined approaches delineate a specific process for combining results from several methods. While several assays for this purpose are already described in accepted test guidelines [14], compared to these, the EASA relies on a standard plate reader as compared to liquid chromatography instrumentation, increasing its potential to be used in a wider range of laboratory settings. Additionally, the assay can run up to seven chemicals in triplicate in one day, providing higher throughput than the other KE1-based assays. For this reason, a consortium of U.S. government and U.S. government contract laboratories undertook this validation study of the EASA to determine whether its performance supports inclusion into TG 442C and potential future evaluation as a KE1 assay for defined approaches (e.g., OECD Guideline 497).

The current validation study focused on optimizing the protocol and evaluating the transferability of the EASA, including assessing its WLR and BLR. OECD performance standards for KE1 assays prescribe benchmarks of at least 80% for both WLR and BLR [15,16]. The EASA met both benchmarks easily, achieving 96% WLR and 85% BLR. This suggests that the study achieved its goal of developing a robust protocol that can be readily transferred to appropriately staffed and equipped laboratories.

### 4.1. Predictive Capacity

A key element of currently accepted approaches to validation of non-animal tests is determining how well the approach agrees with the outcome of the standard animal test, as such tests are often referenced in regulations. This validation study compared predictions of skin sensitization hazard classifications based on EASA data relative to those based on LLNA data and, where available, predictive human patch test data. Accuracy, sensitivity, and specificity of the EASA were calculated as described in Section 2.4 relative to both LLNA and human reference data.

As evaluated against the LLNA, the overall sensitivity of the EASA was 87%, its specificity was 76%, and its accuracy was 83%. While the EASA exceeded the thresholds for sensitivity and accuracy provided in OECD performance standards for TG 442C assays, its specificity did not meet the 80% threshold of the performance standards [15]. As noted above, specificity evaluates the ability of an assay to correctly classify a substance as negative for the endpoint of interest relative to reference data. However, two of the nonsensitizers tested in this assay proved to be challenging. Salicylic acid was found to induce autofluorescence in the PDA assay, resulting in inconclusive calls in three of the four laboratories. This interference highlights limitations of the assay with respect to probe interference. Other challenging nonsensitizers tested in the study were benzyl alcohol and methyl salicylate. Benzyl alcohol has been historically difficult to characterize with respect to its skin sensitization potential. While the compound is a nonsensitizer based on LLNA data, it yielded positive results in human predictive patch tests and conflicting results when tested in the DPRA. In the current study, results of testing benzyl alcohol were flagged as potential false positives, but the ultimate call for the chemical was positive. However, a future consideration may be how to more specifically characterize and address potential false positive flags for chemicals, beyond just an alert. It is worth noting that benzyl alcohol was replaced as a chemical for the performance standards in the 2022 update [16]. Methyl salicylate also yielded discordant results among methods: it is a nonsensitizer in both the LLNA and human patch tests but has returned positive results in the DPRA and was identified as positive in the EASA by all participating laboratories (Table 4). Methyl salicylate was also flagged for interference in the EASA PDA assay, indicating that this may be an incompatibility with the method itself. Example outputs for both benzyl alcohol and methyl salicylate are provided in Supplemental Table S8.

While evaluating predictivity relative to the standard animal test may be of interest for regulatory compliance, evaluating predictivity relative to human data is of interest for human health risk assessment. Relative to our human data set, the EASA achieved an overall sensitivity of 81%, but its accuracy of 79% and specificity of 76% failed to meet the performance standard goals [15]. It is important to note that the specificity metric was based on evaluation of five nonsensitizers. Two of these were dihydroeugenol and methyl salicylate, which are challenging to characterize as discussed above. Further studies with a broader set of human reference data could be valuable to fully characterize the human predictivity of the EASA.

### 4.2. Statistical Testing in EASA

The EASA is unique from other skin sensitization assays in that it relies on the determination of statistical significance via a modified *t*-test, whereas other assays utilize static thresholds that must be exceeded (such as the percent depletion exceeding 8.32% for a positive prediction in the DPRA). There are advantages to both approaches. However, it is worth noting specifically that the statistical approach has the ability to account for variability in the method on a plate-by-plate basis, thereby flagging when something is potentially wrong with a plate or reagents (e.g., volatile chemicals, solubility issues, expired reagents). For example, testing in Laboratory 4 yielded false positive results in one run for isopropanol with a depletion of 1.7% and false negative results for 4-methylamino phenol hemisulfate with a depletion of 34.8%. Subsequent testing within the QT yielded two negative results for isopropanol and two positive results for 4-methylamino phenol hemisulfate, leading to correct overall calls in agreement with the reference data. The discordant results were flagged, highlighting potential issues with that run. Incorporation of flags as part of the decision process may be helpful in revealing questionable results that could need additional confirmatory testing.

### 4.3. Limitations

A limitation of the EASA that it shares with other KE1 assays is its lack of metabolic capacity [12,14]. The prohapten dihydroeugenol is a moderate sensitizer in the LLNA but has returned conflicting results in the DPRA and ADRA. This is likely because metabolic activation is necessary for production of the reactive electrophile that ultimately leads to sensitization. It is worth nothing that dihydroeugenol was removed from the 2022 update to the performance standards and is also negative in a human maximization test, further supporting that testing of this chemical in the EASA would be expected to be problematic [16,20].

It is important to note that the laboratory phase of this validation study was conducted prior to the publication of updated OECD performance standards for KE1 assays [16]. The revised performance standards added new chemicals to the list of reference chemicals and removed others, as discussed previously. Additional testing of the new chemicals on the list may be beneficial.

### 4.4. Lessons Learned

The challenges recounted in the Results section are worth considering in planning future validation studies. The authors of this paper observed variations in plate reader sensitivity, temperature sensitivity, and fluorescence background in different lots of assay plates, which speaks to the value of a proactive evaluation of potential sources of variability in planning a validation study. Petersen et al. (2022) describes an approach to analyzing sources of technical variability in an assay as part of a larger framework for refining a protocol using basic quality tools [23]. The reagent expiration issues encountered highlight the importance of monitoring reagent expiration dates as a routine part of laboratory operations and completing studies, if possible, before reagents expire. Reagents are assessed for concordance with QC parameters, and if any are found to be out of specification, new QC parameters are established specifically to the current lot and time period. These steps are particularly useful if circumstances force a long gap in testing, as was experienced during this study as a result of the COVID-19 pandemic.

## 5. Conclusions

In summary, this validation study supports the utility of the EASA method to predict chemical protein reactivity, which addresses KE1 of the skin sensitization adverse outcome pathway. The study also supports future evaluation of the EASA as an additional method within OECD TG 442C and its use as a KE1 assay in defined approaches (Guideline 497) to discriminate between skin sensitizers and nonsensitizers for hazard classification and labeling and potency categorization according to the United Nations Globally Harmonized System of Classification and Labelling of Chemicals [24]. The authors of this paper acknowledge that additional work to support such an assessment may be of value, and there are additional considerations to that end. First, it could be valuable for subsequent testing to address the new chemical list in the updated performance standards for TG 442C to determine how data from these chemicals affects performance of the assay. Secondly, the authors encourage exploration of a decision process for how to handle potential outcomes flagged as potential false positive/negative results, describing when additional testing could be useful. The expanded dataset included in [12], should also be leveraged for these evaluations.

## Figures and Tables

**Table 1:** Reference chemicals for the EASA validation study. Modified and reprinted with permission from [17].

Chemical Name	CAS No.	LLNA EC3 (%)	In Vivo Prediction	DPRA Results	ADRA Results	Study Phase
Lauryl gallate	1166-52-5	0.3	Strong sensitizer	Pos	Pos	WLR/BLR
Chloramine trihydrate	127-65-1	0.4	Strong sensitizer	Pos	Pos	WLR/BLR
4-(Methylamino) phenol, hemisulfate	55-55-0	0.8	Strong sensitizer	Pos	Pos	WLR/BLR
2-Mercaptobenzothiazole	149-30-4	1.7	Moderate sensitizer	Pos	Pos	WLR/BLR
Benzyl salicylate	118-58-1	2.9	Moderate sensitizer	Pos/Neg	Neg	WLR/BLR
Cinnamaldehyde	14371-10-9 (104-55-2)	3	Moderate sensitizer	Pos	Pos	WLR/BLR
Imidazolidinyl urea	39236-46-9	24	Moderate sensitizer	Pos	Pos	WLR/BLR
Ethyl acrylate	140-88-5	28	Weak sensitizer	Pos	Pos	WLR/BLR
Salicylic acid	69-72-7	-	Nonsensitizer	Pos/Neg	Neg	WLR/BLR
Benzyl alcohol	100-51-6	-	Nonsensitizer	Pos/Neg	Neg	WLR/BLR
Glycerol	56-81-5	-	Nonsensitizer	Neg	Neg	WLR/BLR
Isopropanol	67-63-0	-	Nonsensitizer	Neg	Neg	WLR/BLR
p-Benzoquinone	106-51-4	0.0099	Extreme sensitizer	Pos	Pos	BLR
Dihydroeugenol	2785-87-7	6.8	Moderate sensitizer	Pos/Neg	Pos/Neg	BLR
Palmitoyl chloride	112-67-4	8.8	Moderate sensitizer	Pos	Pos	BLR
Farnesal	19317-11-4 (502-67-0)	12	Weak sensitizer	Pos	Pos	BLR
Benzyl cinnamate	103-41-3	18	Weak sensitizer	Neg	Neg	BLR
Dimethyl isophthalate	1459-93-4	-	Nonsensitizer	Neg	Neg	BLR
Methyl salicylate	119-36-8	-	Nonsensitizer	Pos/Neg	Neg	BLR
4-Aminobenzoic acid	150-13-0	-	Nonsensitizer	Neg	Neg	BLR

Abbreviations: BLR = between-laboratory reproducibility; CAS = Chemical Abstracts Service; EC3 = concentration inducing a stimulation index of 3; Neg = negative; Pos = positive; WLR = within-laboratory reproducibility.

**Table 2:** Results of within-laboratory reproducibility testing. Modified and reprinted with permission from [17].

Chemical Name	In Vivo Prediction	EASA Result Laboratory 1	EASA Result Laboratory 2	EASA Result Laboratory 3	EASA Result Laboratory 4	EASA Result Consensus
Lauryl gallate	Strong sensitizer	Pos	Pos	Pos	Pos	Pos
Chloramine trihydrate	Strong sensitizer	Pos	Pos	Pos	Pos	Pos
4-(Methylamino) phenol, hemisulfate	Strong sensitizer	Pos	Pos	Pos	Pos	Pos
2-Mercaptobenzothiazole	Moderate sensitizer	Pos	Pos	Pos	Pos	Pos
Benzyl salicylate	Moderate sensitizer	Pos	Pos	Pos	Pos	Pos
Cinnamaldehyde	Moderate sensitizer	Pos	Pos	Pos	Pos	Pos
Imidazolidinyl urea	Moderate sensitizer	Pos	Pos	Pos	Pos	Pos
Ethyl acrylate	Weak sensitizer	Neg	Neg	Neg	Neg	Neg
Salicylic acid	Nonsensitizer	Inc	Neg	Inc	Inc	Inc
Benzyl alcohol	Nonsensitizer	Neg	Pos	Neg	Pos	Inc
Glycerol	Nonsensitizer	Neg	Neg	Neg	Neg	Neg
Isopropanol	Nonsensitizer	Neg	Neg	Neg	Neg	Neg

Abbreviations: Inc—inconclusive; Neg = negative; Pos = positive. In cases where the majority of laboratories had an inconclusive result or two laboratories had both positive and negative results, the consensus result was inconclusive.

**Table 3:** Results of between-laboratory reproducibility testing and comparison to reference data. Reprinted with permission from [17].

Chemical Name	In Vivo Prediction (LLNA)	EASA Lab. 1	EASA Lab. 2	EASA Lab. 3	EASA Lab. 4	EASA Consensus	Human Ref.	DPRA Ref.	ADRA Ref.
Lauryl gallate	Strong sensitizer	Pos	Pos	Pos	Pos	Pos	Pos ^1^	Pos	Pos
Chloramine trihydrate	Strong sensitizer	Pos	Pos	Pos	Pos	Pos	N/A	Pos	Pos
4-(Methylamino) phenol, hemisulfate	Strong sensitizer	Pos	Pos	Pos	Pos	Pos	Pos ^1^	Pos	Pos
2-Mercaptobenzothiazole	Moderate sensitizer	Pos	Pos	Pos	Pos	Pos	Pos ^2^	Pos	Pos
Benzyl salicylate	Moderate sensitizer	Pos	Pos	Pos	Pos	Pos	Pos/ Neg ^3^	Pos/ Neg	Neg
Cinnamaldehyde	Moderate sensitizer	Pos	Pos	Pos	Pos	Pos	Pos ^2^	Pos	Pos
Imidazolidinyl urea	Moderate sensitizer	Pos	Pos	Pos	Pos	Pos	Pos ^2^	Pos	Pos
Ethyl acrylate	Weak sensitizer	Neg	Neg	Neg	Neg	Neg	Pos ^2^	Pos	Pos
Salicylic acid	Nonsensitizer	Inc	Neg	Inc	Inc	Inc	Neg ^3^	Pos/ Neg	Neg
Benzyl alcohol	Nonsensitizer	Neg	Pos	Neg	Pos	Inc	Pos ^3^	Pos/ Neg	Neg
Glycerol	Nonsensitizer	Neg	Neg	Neg	Neg	Neg	Neg ^3^	Neg	Neg
Isopropanol	Nonsensitizer	Neg	Neg	Neg	Neg	Neg	Neg ^1^	Neg	Neg
p-Benzoquinone	Extreme sensitizer	Pos	Pos	Pos	Pos	Pos	N/A	Pos	Pos
Dihydroeugenol	Moderate sensitizer	Neg	Pos	Neg	Neg	Neg	Neg ^3^	Pos/ Neg	Pos/ Neg
Palmitoyl chloride	Moderate sensitizer	Pos	Pos	Pos	Pos	Pos	N/A	Pos	Pos
Farnesal	Weak sensitizer	Pos	Pos	Pos	Pos	Pos	N/A	Pos	Pos
Benzyl cinnamate	Weak sensitizer	Pos	Pos	Pos	Pos	Pos	Pos ^1^	Neg	Neg
Dimethyl isophthalate	Nonsensitizer	Neg	Neg	Neg	Neg	Neg	N/A	Neg	Neg
Methyl salicylate	Nonsensitizer	Pos	Pos	Pos	Pos	Pos	Neg ^3^	Pos/ Neg	Neg
4-Aminobenzoic acid	Nonsensitizer	Neg	Neg	Neg	Neg	Neg	Neg ^3^	Neg	Neg

Abbreviations: Inc = inconclusive; Lab. = laboratory; N/A = human reference data not available; Neg = negative; Pos = positive, Pos/Neg = no definitive call from reference data. Human reference data source: [17].

**Table 4:** Comparison of EASA calls to reference test calls. Modified and reprinted with permission from [17].

Test Chemical	Mechanism ^†^	EASA	LLNA	DPRA	ADRA	Potency ^†^
p-Benzoquinone	Michael acceptor	Pos	Pos	Pos	Pos	Extreme
Lauryl gallate	Pre-hapten Michael acceptor	Pos	Pos	Pos	Pos	Strong
Chloramine trihydrate	Acylation	Pos	Pos	Pos	Pos	Strong
4-(Methylamino) phenol, hemisulfate	Pre-hapten Michael acceptor	Pos	Pos	Pos	Pos	Strong
2-Mercaptobenzothiazole	SN2, acylation	Pos	Pos	Pos	Pos	Moderate
Benzyl salicylate	SN2, acylation	Pos	Pos	Pos/Neg	Neg	Moderate
Cinnamaldehyde *	Michael acceptor	Pos	Pos	Pos	Pos	Moderate
Imidazolidinyl urea	Acylation	Pos	Pos	Pos	Pos	Moderate
Dihydroeugenol *	Pro-hapten, SN2, Michael acceptor	Pos/Neg	Pos	Pos/Neg	Pos/Neg	Moderate
Palmitoyl chloride	Acylation	Pos	Pos	Pos	Pos	Moderate
Ethyl acrylate	Michael acceptor	Neg	Pos	Pos	Pos	Weak
Farnesal	Schiff base	Pos	Pos	Pos	Pos	Weak
Benzyl cinnamate	SN2, Acylation	Pos	Pos	Neg	Neg	Weak
Salicylic acid	Non-reactive	Neg/Inc	Neg	Pos/Neg	Neg	Nonsensitizer
Benzyl alcohol *	Non-reactive	Pos/Neg	Neg	Pos/Neg	Neg	Nonsensitizer
Glycerol	Non-reactive	Neg	Neg	Neg	Neg	Nonsensitizer
Isopropanol	Non-reactive	Neg	Neg	Neg	Neg	Nonsensitizer
Dimethyl isophthalate	Non-reactive	Neg	Neg	Neg	Neg	Nonsensitizer
Methyl salicylate	Non-reactive	Pos	Neg	Pos/Neg	Neg	Nonsensitizer
4-Aminobenzoic acid	Non-reactive	Neg	Neg	Neg	Neg	Nonsensitizer

Abbreviations: Neg = negative; Pos = positive; Inc = inconclusive. ^†^ As described in OECD, 2019 [15]. * Chemicals replaced in 2022 OECD performance standards update [16]. Yellow fill indicates where classifications based on EASA results do not match LLNA reference classifications; green fill indicates where classifications based on existing TG 442C methods do not match LLNA reference classifications.

## Data Availability

The original data presented in the study are openly available at https://doi.org/10.22427/NICEATM-3 and https://doi.org/10.22427/NICEATM-DATA-NICEATM-3.

## Supplementary Materials

The following supporting information can be downloaded at: https://www.mdpi.com/article/10.3390/toxics14060511/s1, Supplemental Figures: Supplemental Figure S1. Evaluation of trends in the negative control data for the absorbance (NBT) and fluorescence (PDA) among pipetting steps among laboratories; Supplemental Figure S2. Chamber temperature variations for Laboratory 3. Supplemental Tables: Supplemental Table S1. Evaluation of probe concentration using half or twice nominal concentration for seven reference chemicals; Supplemental Table S2. Laboratory 1 baseline QC parameters; Supplemental Table S3. Laboratory 2 baseline QC parameters; Supplemental Table S4. Laboratory 3 baseline QC parameters; Supplemental Table S5. Laboratory 4 baseline QC parameters; Supplemental Table S6. Replicate QC calls per laboratory; Supplemental Table S7. Individual laboratory results for between-laboratory reproducibility; Supplemental Table S8. Example output of methyl salicylate and benzyl alcohol.

## Abbreviations

The following abbreviations are used in this manuscript:

ACN	Acetonitrile
ADRA	Amino acid derivative reactivity assay
BLR	Between-laboratory reproducibility
COV	Coefficient of variation
CPSC	U.S. Consumer Product Safety Commission
DPRA	Direct peptide reactivity assay
EASA	Electrophilic allergen screening assay
EPA	U.S. Environmental Protection Agency
FN	False negative
FP	False positive
IC_50_	Half-maximal inhibitory concentration
Inc	Inconclusive
KE	Key event
Lab.	Laboratory
LLNA	Murine local lymph node assay
N/A	Data not available
NBT	4-Nitrobenzenethiol
NC	Negative control
Neg	Negative
NICEATM	National Toxicology Program (NTP) Interagency Center for the Evaluation of Alternative Toxicological Methods
NIST	National Institute of Standards and Technology
NTP	National Toxicology Program
OECD	Organisation for Economic Co-operation and Development
PC	Positive control
PDA	Pyridoxylamine hydrochloride
Pos	Positive
QC	Quality control
QT	Qualified test
SS	Solvent system
SOP	Standard operating procedure
TC	Test chemical
TG	Test Guideline
TN	True negative
TP	True positive
WLR	Within-laboratory reproducibility
