# Supplementary material for: Validation of the Electrophilic Allergen Screening Assay for Detection of Key Event 1 of the Skin Sensitization Adverse Outcome Pathway
Source: Toxics. 2026 Jun 11;14(6):511. doi: 10.3390/toxics14060511 (PMC13307052; doi:10.3390/toxics14060511)
Supplement: Supplementary file 1 [file toxics-14-00511-s001.zip › toxics-4275763-supplementary.pdf]

Supplemental Materials for "Validation of the Electrophilic Allergen Screening Assay for Detection of Key Event 1 of the Skin Sensitization Adverse Outcome Pathway"

Emily N. Reinke[1,2*], Elijah J. Petersen[3*], John Gordon[4], Rick Uhl[4], Valerie H. Adams[5], Diego Rua[6], Victor J. Johnson[7], Gary Burleson[7], Judy Strickland[1], Robert Gutierrez[3], Catherine S. Sprankle[1,2], Tripp LaPratt[1,2], David M. Lehmann[8], Dori Germolec[9], Nicole C. Kleinstreuer[10]

[1] Inotiv, Inc., Morrisville, NC 27560 USA
[2] GDIT, Inc., Falls Church, VA, 22042, USA
[3] National Institute of Standards and Technology, Gaithersburg, MD 20899, USA
[4] U.S. Consumer Product Safety Commission, Rockville, MD 20850, USA
[5] Defense Centers for Public Health—Aberdeen, Aberdeen, MD 21005, USA
[6] DRX2 Consulting LLC, Washington, DC 20001, USA
[7] Burleson Research Technologies, Inc. Morrisville, NC 27506, USA
[8] Intertox, LLC, Seattle, WA 98101, USA; dlehmann@intertox.com
[9] National Institute of Environmental Health Sciences, Durham, NC 27709, USA; germolec@niehs.nih.gov
[10] Division of Program Coordination, Planning, and Strategic Initiatives, National Institutes of Health, Bethesda, MD 20892, USA

* Correspondence: emily.reinke@gdit.com; elijah.petersen@nist.gov

Index:

Supplemental Figures:

Supplemental Figure S1. Evaluation of trends in the negative control data for the absorbance (NBT) and fluorescence (PDA) among pipetting steps among laboratories. 3
Supplemental Figure S2. Chamber Temperature Variations for Laboratory 3 4

Tables:

Supplemental Table S1. Evaluation of probe concentration using half or twice nominal concentration for seven reference chemicals........5
Supplemental Table S2. Laboratory 1 baseline QC parameters 6
Supplemental Table S3. Laboratory 2 baseline QC parameters 7
Supplemental Table S4. Laboratory 3 baseline QC parameters 8
Supplemental Table S5. Laboratory 4 baseline QC parameters 9
Supplemental Table S6. Replicate QC calls per laboratory........10

Supplemental Table S7. Individual Laboratory Results for Between-Laboratory Reproducibility 11
Supplemental Table S8. Example output of methyl salicylate and benzyl alcohol 12

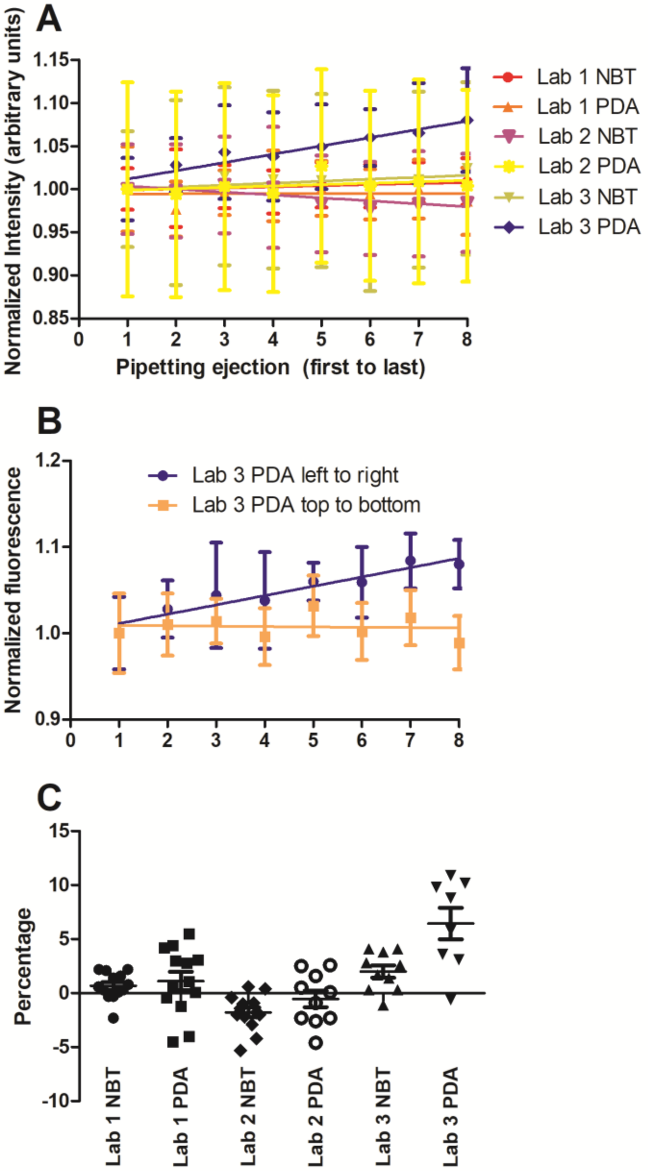

Supplemental Figure S1. Evaluation of trends in the negative control data for the absorbance (NBT) and fluorescence (PDA) among pipetting steps among laboratories.

Data and error bars represent the mean and standard deviation of at least eight plates (A and B). The lines represent linear regression fits (A and B). The linear regression for Lab 3 PDA showed a statistical difference compared to 0 for left to right ($p = 0.0003$), but not top to bottom ($p = 0.85$). Comparison of the NC values in the columns for the test samples (columns 6 to 9) to those in the first column used for pipetting the NC (column 2); each data point represents a separate plate (C). This data shows that the results in parts A and B reflect a trend observed among multiple plates.

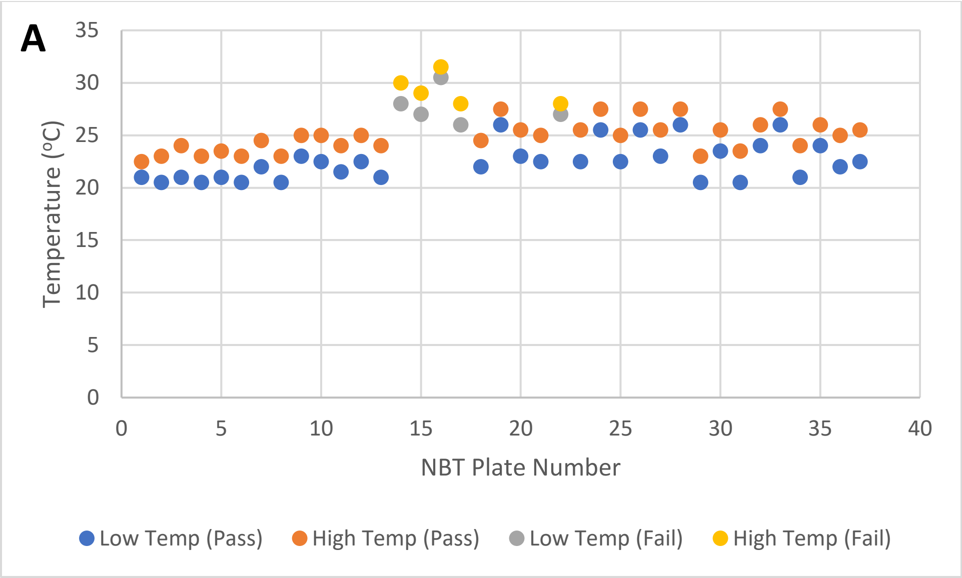

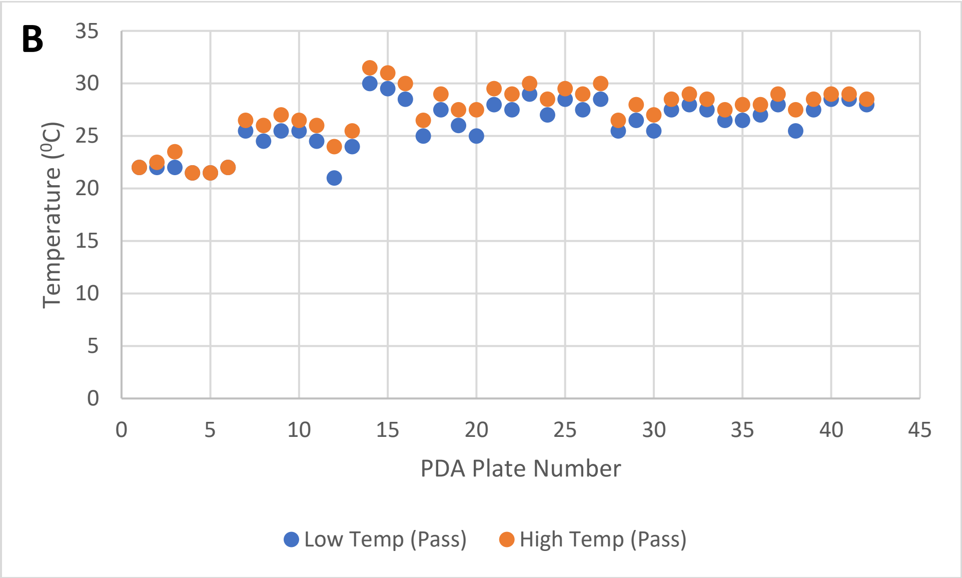

Supplemental Figure S2. Chamber Temperature Variations for Laboratory 3

Data in represents that minimum and maximum chamber temperatures during an assay run, across all plates in the Laboratory 3 validation study. The top figure (**A**) shows the NBT plates, orange and blue dots indicate passing plates, grey and yellow dots indicate plates that failed the IC_{50} estimate for QC. The bottom figure (**B**) shows all PDA plates, there were no failed plates for the IC_{50} estimate for QC.

Supplemental Table S1. Evaluation of probe concentration using half or twice nominal concentration for seven reference chemicals

**Probe concentration**	**Dimethyl isophthalate**	**Methyl salicylate**	**Dihydroeugenol**	**Lauryl gallate**	**Isopropanol**	**Chloramine T trihydrate**	**Ethyl acrylate**
Normal (NBT)	NNN	NNN	NNN	NNP	NNN	PPP	NNN
Normal (PDA)	NNN	PPP	NPN	PPP	NN	PP	NN
Normal (Overall)	NNN	PPP	NPN	PPP	NN	PP	NN
Half concentration (NBT)	NNN	NNN	NNN	NPP	NNN	PPP	NNN
Half concentration (PDA)	NN	PP	NPN	NIP	NN	PP	NN
Half concentration (Overall)	NN	PP	NPN	NPP	NN	PP	NN
2x concentration (NBT)	NN	NN	NN	PP	NN	PP	NN
2x concentration (PDA)	NN	PP	NN	PP	NN	PP	NN
2x concentration (Overall)	NN	PP	NN	PP	NN	PP	NN
**Qualified test**							
Normal	N	P	N	P	N	P	N
Half concentration	N	P	N	P	N	P	N
2x concentration	N	P	N	P	N	P	N
**Concordance**	100 %	100 %	100 %	100 %	100 %	100 %	100 %

N = negative, P = positive, I = inconclusive; multiple entries within a cell indicate separate independent runs for that probe. Qualified Test indicates the overall call related to the concordance from 2 of 2 or 2 of 3 independent runs.

Supplemental Table S2. Laboratory 1 baseline QC parameters

NBT absorbance QC report data

	NC/PC Blank	NC	NC COV	PC Midpoint	PC Forecast IC_{50} (mmol/L)	Benzyl bromide (PC) 0.600 mmol/L	Benzyl bromide (PC) 0.300 mmol/L	Benzyl bromide (PC) 0.150 mmol/L	Benzyl bromide (PC) 0.075 mmol/L	Benzyl bromide (PC) 0.038 mmol/L	Benzyl bromide (PC) 0.019 mmol/L	Benzyl bromide (PC) 0.009 mmol/L
Upper Limit	0.072	0.863	3.48 %	54.1 %	0.1131	99.3 %	97.1 %	78.6 %	52.5 %	29.2 %	17.7 %	10.0 %
Lower Limit	0.067	0.559	-0.94 %	50.4 %	0.0848	96.1 %	87.4 %	64.5 %	37.4 %	21.5 %	10.1 %	3.3 %

PDA fluorescence QC report data

	NC/PC Blank	NC	NC COV	PC Midpoint	PC Forecast IC_{50} (mmol/L)	Glutaralde-hyde (PC) 0.0200 mmol/L	Glutaralde-hyde (PC) 0.0100 mmol/L	Glutaralde-hyde (PC) 0.0050 mmol/L	Glutaralde-hyde (PC) 0.0025 mmol/L	Glutaralde-hyde (PC) 0.0013 mmol/L	Glutaralde-hyde (PC) 0.0006 mmol/L	Glutaralde-hyde (PC) 0.0003 mmol/L
Version 1 Upper Limit	5416.3	53487.5	6.78 %	57.8 %	0.0035	103.9 %	100.6 %	95.6 %	65.2 %	36.7 %	20.8 %	15.1 %
Version 1 Lower Limit	4443.8	32206.6	-1.74 %	44.7 %	0.0022	91.1 %	88.7 %	69.9 %	32.7 %	12.4 %	1.5 %	-5.6 %
Version 2 Upper Limit	16107.5	55741.8	4.98 %	52.6 %	0.0031	99.8 %	97.7 %	88.4 %	51.8 %	32.1 %	15.8 %	9.6 %
Version 2 Lower Limit	10065.8	33521.7	-1.45 %	50.0 %	0.0027	95.2 %	91.7 %	74.2 %	43.9 %	17.0 %	7.4 %	1.0 %
Version 3 Upper Limit	13342.6	55377.2	10.91 %	61.1 %	0.0032	104.0 %	100.4 %	91.7 %	84.7 %	51.0 %	29.8 %	25.8 %
Version 3 Lower Limit	9357.9	24926.4	-4.23 %	47.5 %	0.0016	93.3 %	92.6 %	86.8 %	39.5 %	16.7 %	4.5 %	-5.0 %

Abbreviations: COV = coefficient of variation; IC_{50} = half-maximal inhibitory concentration; NBT = 4-nitrobenzenthiol; NC = negative control; PC = positive control; PDA = pyridoxylamine; QC = quality control.

Supplemental Table S3. Laboratory 2 baseline QC parameters

NBT absorbance QC report data

	NC/PC Blank	NC	NC COV	PC Midpoint	PC Forecast IC_{50} (mmol/L)	Benzyl bromide (PC) 0.600 mmol/L	Benzyl bromide (PC) 0.300 mmol/L	Benzyl bromide (PC) 0.150 mmol/L	Benzyl bromide (PC) 0.075 mmol/L	Benzyl bromide (PC) 0.038 mmol/L	Benzyl bromide (PC) 0.019 mmol/L	Benzyl bromide (PC) 0.009 mmol/L
Upper Limit	0.093	0.686	4.2 %	59.0 %	0.170	101.4 %	103.7 %	94.3 %	77.5 %	48.9 %	32.1 %	19.9 %
Lower Limit	0.067	0.318	-1.6 %	44.7 %	0.009	93.8 %	87.3 %	65.1 %	30.4 %	12.9 %	1.5 %	-3.8 %

PDA fluorescence QC report data

	NC/PC Blank	NC	NC COV	PC Midpoint	PC Forecast IC_{50} (mmol/L)	Glutaraldehyde (PC) 0.0200 mmol/L	Glutaraldehyde (PC) 0.0100 mmol/L	Glutaraldehyde (PC) 0.0050 mmol/L	Glutaraldehyde (PC) 0.0025 mmol/L	Glutaraldehyde (PC) 0.0013 mmol/L	Glutaraldehyde (PC) 0.0006 mmol/L	Glutaraldehyde (PC) 0.0003 mmol/L
Upper Limit	41.4	316.6	7.6 %	63.1 %	0.008	106.5 %	95.6 %	65.1 %	38.3 %	22.0 %	21.8 %	26.4 %
Lower Limit	16.5	15.3	-2.7 %	34.8 %	0.003	83.7 %	58.0 %	19.3 %	0.7 %	-5.2 %	-14.5 %	-20.8 %

Abbreviations: COV = coefficient of variation; IC_{50} = half-maximal inhibitory concentration; NBT = 4-nitrobenzenthiol; NC = negative control; PC = positive control; PDA = pyridoxylamine; QC = quality control.

Supplemental Table S4. Laboratory 3 baseline QC parameters

NBT absorbance QC report data

	NC/PC Blank	NC	NC COV	PC Midpoint	PC Forecast IC_{50} (mmol/L)	Benzyl bromide (PC) 0.600 mmol/L	Benzyl bromide (PC) 0.300 mmol/L	Benzyl bromide (PC) 0.150 mmol/L	Benzyl bromide (PC) 0.075 mmol/L	Benzyl bromide (PC) 0.038 mmol/L	Benzyl bromide (PC) 0.019 mmol/L	Benzyl bromide (PC) 0.009 mmol/L
Upper Limit	0.082	1.116	5.2 %	56.8 %	0.097	100.3 %	100.4 %	92.9 %	73.3 %	45.9 %	25.9 %	17.5 %
Lower Limit	0.062	0.365	-2.3 %	49.7 %	0.066	96.9 %	93.1 %	72.4 %	38.8 %	18.0 %	8.2 %	-0.7 %

PDA fluorescence QC report data

	NC/PC Blank	NC	NC COV	PC Midpoint	PC Forecast IC_{50} (mmol/L)	Glutaraldehyde (PC) 0.0200 mmol/L	Glutaraldehyde (PC) 0.0100 mmol/L	Glutaraldehyde (PC) 0.0050 mmol/L	Glutaraldehyde (PC) 0.0025 mmol/L	Glutaraldehyde (PC) 0.0013 mmol/L	Glutaraldehyde (PC) 0.0006 mmol/L	Glutaraldehyde (PC) 0.0003 mmol/L
Upper Limit	3460104.9	18550819.3	14.5 %	108.2 %	0.015	111.7 %	109.0 %	117.9 %	162.3 %	179.8 %	137.6 %	82.7 %
Lower Limit	242060.1	3268105.4	-6.8 %	7.6 %	-0.015	81.2 %	83.8 %	68.6 %	3.1 %	-51.1 %	-59.1 %	-35.1 %

Abbreviations: COV = coefficient of variation; IC_{50} = half-maximal inhibitory concentration; NBT = 4-nitrobenzenthiol; NC = negative control; PC = positive control; PDA = pyridoxylamine; QC = quality control.

Supplemental Table S5. Laboratory 4 baseline QC parameters

NBT absorbance QC report data

	NC/PC Blank	NC	NC COV	PC Midpoint	PC Forecast IC_{50} (mmol/L)	Benzyl bromide (PC) 0.600 mmol/L	Benzyl bromide (PC) 0.300 mmol/L	Benzyl bromide (PC) 0.150 mmol/L	Benzyl bromide (PC) 0.075 mmol/L	Benzyl bromide (PC) 0.038 mmol/L	Benzyl bromide (PC) 0.019 mmol/L	Benzyl bromide (PC) 0.009 mmol/L
Upper Limit	0.099	0.886	6.8 %	55.6 %	0.147	100.6 %	99.3 %	93.0 %	55.5 %	34.0 %	22.6 %	12.0 %
Lower Limit	0.057	0.386	-0.9 %	49.9 %	0.062	94.8 %	86.5 %	47.4 %	29.3 %	11.5 %	1.0 %	3.5 %

PDA fluorescence QC report data

	NC/PC Blank	NC	NC COV	PC Midpoint	PC Forecast IC_{50} (mmol/L)	Glutaraldehyde (PC) 0.0200 mmol/L	Glutaraldehyde (PC) 0.0100 mmol/L	Glutaraldehyde (PC) 0.0050 mmol/L	Glutaraldehyde (PC) 0.0025 mmol/L	Glutaraldehyde (PC) 0.0013 mmol/L	Glutaraldehyde (PC) 0.0006 mmol/L	Glutaraldehyde (PC) 0.0003 mmol/L
Upper Limit	1277827.0	10919037.1	12.8 %	65.0 %	0.008	97.5 %	100.8 %	120.5 %	57.4 %	50.6 %	34.5 %	34.8 %
Lower Limit	597433.3	3685662.5	-0.1 %	39.2 %	0.000	92.3 %	79.3 %	10.3 %	13.1 %	-3.6 %	-1.1 %	-12.8 %

Abbreviations: COV = coefficient of variation; IC_{50} = half-maximal inhibitory concentration; NC = negative control; NBT = 4-nitrobenzenthiol; PC = positive control; PDA = pyridoxylamine; QC = quality control.

Supplemental Table S6. Replicate QC calls per laboratory

Chemical Name	Lab 1			Lab 2			Lab 3			Lab 4		
	QT 1	QT 2	QT 3	QT 1	QT 2	QT 3	QT 1	QT 2	QT 3	QT 1	QT 2	QT 3
Lauryl gallate	Pos	Pos	Pos	Pos	Pos	Pos	Pos	Pos	Pos	Pos	Pos	Pos
Chloramine trihydrate	Pos	Pos	Pos	Pos	Pos	Pos	Pos	Pos	Pos	Pos	Pos	Pos
4-(Methylamino) phenol, hemisulfate	Pos	Pos	Pos	Pos	Pos	Pos	Pos	Pos	Pos	Pos	Pos	Pos
2-Mercaptobenzothiazole	Pos	Pos	Pos	Pos	Pos	Pos	Pos	Pos	Pos	Pos	Pos	Pos
Benzyl salicylate	Pos	Pos	Pos	Pos	Pos	Pos	Pos	Pos	Pos	Pos	Pos	Pos
Cinnamaldehyde	Pos	Pos	Pos	Pos	Pos	Pos	Pos	Pos	Pos	Pos	Pos	Pos
Imidazolidinyl urea	Pos	Pos	Inc	Pos	Pos	Pos	Pos	Pos	Pos	Pos	Pos	Pos
Ethyl acrylate	Neg	Neg	Neg	Neg	Neg	Neg	Neg	Neg	Neg	Neg	Neg	Neg
Salicylic acid	Inc	Inc	Inc	Neg	Neg	Neg	Inc	Inc	Inc	Inc	Pos	Inc
Benzyl alcohol	Neg	Neg	Pos	Pos	Pos	Pos	Neg	Neg	Neg	Pos	Pos	Pos
Glycerol	Neg	Neg	Neg	Neg	Neg	Neg	Neg	Neg	Neg	Neg	Neg	Neg
Isopropanol	Neg	Neg	Neg	Neg	Neg	Neg	Neg	Neg	Neg	Neg	Neg	Neg

QT = qualified test, Pos = positive, Neg = negative, Inc = Inconclusive.

Supplemental Table S7. Individual Laboratory Results for Between-Laboratory Reproducibility

Chemical Name	LLNA Reference	Lab 1	Lab 2	Lab 3	Lab 4
Lauryl Gallate	Strong	Pos	Pos	Pos	Pos
Chloramine T trihydrate	Strong	Pos	Pos	Pos	Pos
Metol (4-methylamino phenol)	Strong	Pos	Pos	Pos	Pos
2-Mercaptobenzothiazole	Moderate	Pos	Pos	Pos	Pos
Benzyl salicylate	Moderate	Pos	Pos	Pos	Pos
Cinnamaldehyde	Moderate	Pos	Pos	Pos	Pos
Imidazolidynl urea	Moderate	Pos	Pos	Pos	Pos
Ethyl acrylate	Weak	Neg	Neg	Neg	Neg
Salicylic acid	Nonsensitizer	Inc	Neg	Inc	Inc
Benzyl alcohol	Nonsensitizer	Neg	Pos	Neg	Pos
Glycerol	Nonsensitizer	Neg	Neg	Neg	Neg
Isopropanol	Nonsensitizer	Neg	Neg	Neg	Neg
p-Benzoquinone	Extreme	Pos	Pos	Pos	Pos
Dihydroeugenol	Moderate	Neg	Pos	Neg	Neg
Palmitoyl chloride	Moderate	Pos	Pos	Pos	Pos
Farnesal	Weak	Pos	Pos	Pos	Pos
Benzyl cinnamate	Weak	Pos	Pos	Pos	Pos
Dimethyl isophthalate	Nonsensitizer	Neg	Neg	Neg	Neg
Methyl salicylate	Nonsensitizer	Pos	Pos	Pos	Pos
4-Aminobenzoic acid	Nonsensitizer	Neg	Neg	Neg	Neg

Supplemental Table S8. Example output of methyl salicylate and benzyl alcohol

Methyl Salicylate

Time (min)	% Depletion	t-test Call	PFP	PFN	PHI	Interference
5	62.0 %	Positive	-	-	-	Int
20	62.0 %	Positive	-	-	-	Int
35	63.0 %	Positive	-	-	-	Int
**50**	**66.5 %**	**Positive**	**-**	**-**	**-**	**Int**

Benzyl Alcohol

Time (min)	% Depletion	t-test Call	PFP	PFN	PHI	Interference
5	3.8 %	Positive	R	-	-	P-Int
20	6.4 %	Positive	R	-	-	P-Int
35	7.9 %	Positive	R	-	-	P-Int
**50**	**8.9 %**	**Positive**	**R**	**-**	**-**	**P-Int**

Int = interference, P-Int = potential interference, PFP = potential false positive, PFN = potential false negative, PHI = potential high interference, Notations of "R" in "PFN", "PFP", or "PHI" columns indicate scenarios in which additional testing might be needed to address specific regulatory information requirements.
